# Influence of Conductive Filler Types on the Ratio of Reflection and Absorption Properties in Cement-Based EMI Shielding Composites

**DOI:** 10.3390/ma17194913

**Published:** 2024-10-08

**Authors:** Daeik Jang, Jihoon Park, Woosuk Jang, Jinho Bang, G. M. Kim, Jaesuk Choi, Joonho Seo, Beomjoo Yang

**Affiliations:** 1 Center for Advanced Construction Materials, Department of Civil Engineering, The University of Texas at Arlington, Arlington, TX 76019, USA; jang.daeik@uta.edu; 2 Department of Civil and Environmental Engineering, Korea Advanced Institute of Science and Technology (KAIST), 291 Daehak-ro, Yuseong-gu, Daejeon 34141, Republic of Korea; pjspjh930@kaist.ac.kr; 3 School of Civil Engineering, Chungbuk National University, 1 Chungdae-ro, Seowon-gu, Cheongju 28644, Chungbuk, Republic of Korea; wkdws0312@chungbuk.ac.kr (W.J.); jinhobang@chungbuk.ac.kr (J.B.); 2017024189@chungbuk.ac.kr (J.C.); 4 Mineral Processing & Metallurgy Research Center, Resources Utilization Division, Korea Institute of Geoscience and Mineral Resources, 124 Gwahak-ro, Yuseong-gu, Daejeon 34132, Republic of Korea; k.gm@kigam.re.kr; 5 Applied Science Research Institute, Korea Advanced Institute of Science and Technology (KAIST), 291 Daehak-ro, Yuseong-gu, Daejeon 34141, Republic of Korea

**Keywords:** electromagnetic wave interference, carbon nanotube, carbon fiber, graphene nanoplate, absorption, reflection

## Abstract

The growing importance of electromagnetic interference (EMI) shielding composites in civil engineering has garnered increasing attention. Conductive cement-based composites, incorporating various conductive fillers, such as carbon nanotubes (CNTs), carbon fibers (CFs), and graphene nanoplatelets (GNPs), provide effective solutions due to their high electrical conductivity. While previous studies have primarily focused on improving the overall shielding effectiveness, this research emphasizes balancing the reflection and absorption properties. The experimental results demonstrate an EMI shielding performance exceeding 50 dB, revealing that filler size (nano, micro, or macro) and shape (platelet or fiber) significantly influence both reflection and absorption characteristics. Based on a comprehensive evaluation of the shielding properties, this study highlights the need to consider factors such as reflection versus absorption losses and filler shape or type when optimizing filler content to develop effective cement-based EMI shielding composites.

## 1. Introduction

The rapid advancements in electronic and telecommunication technologies underscore the significance of electromagnetic wave interference (EMI) shielding composites due to the potential health hazards posed by EM waves and their interference with electronic devices [1,2,3,4]. In civil engineering, various infrastructure and construction equipment are vulnerable to EM waves, necessitating the widespread adoption of cement-based EMI shielding composites for protection [5,6,7]. Initially, metallic materials were utilized in the development of these composites; however, their use posed challenges related to corrosion and increased composite weight, limiting their applicability in civil infrastructure [8,9,10]. In recent years, researchers have turned to carbon-based conductive fillers, such as carbon nanotubes (CNTs), carbon fibers (CFs), carbon black (CB), and graphene nanoplatelets (GNPs), to fabricate conductive cement for EMI shielding applications [11,12]. Incorporating such fillers ensures favorable electrical conductivity, enabling the development of cement-based EMI shielding composites with excellent EMI shielding performance. Nam et al. [13] reported that the addition of 1 wt% CNTs to 2.36 mm thick cement composites resulted in a 29 dB EMI shielding performance within the X-band frequency range. Yoon et al. developed conductive cement incorporating 0.2 wt% CNTs and 0.5% CFs, achieving a total shielding effectiveness of 18 dB at 10 GHz input frequency [14]. Additionally, Park et al. [15] demonstrated that a 0.2 vol% of CFs in concrete with a thickness of 10 cm exhibited 40 dB of total shielding effectiveness across a frequency range of 600 to 2000 MHz. Many researchers have explored the synergistic effects of combining different types of conductive fillers with varying sizes and shapes to enhance the electrical conductivity of composites. This synergistic interaction can reduce the required amount of conductive filler while simultaneously improving both electrical conductivity and functional properties [14]. For example, previous studies have demonstrated that the combination of CNTs and CFs can create hierarchical conductive networks within composites, leading to improvements in both electrical conductivity and mechanical properties [16,17]. However, fewer studies have investigated the synergistic effects of these fillers on enhancing the EMI shielding performance of cement composites, particularly in relation to reflection and absorption properties.

It is noteworthy that the total shielding effectiveness of EMI shielding composites comprises both reflection and absorption properties. However, previous research efforts in developing cement-based EMI shielding composites have primarily been aimed at enhancing total shielding effectiveness without thoroughly investigating the balance between reflection and absorption properties. Even though increasing the total shielding effectiveness is desirable, an increase in the reflection property may inadvertently direct EM waves toward other electronic devices or pose health risks to individuals [18]. Therefore, while significant strides have been made in improving the total shielding effectiveness, few studies have focused on examining the ratio of the shielding properties. In this regard, this study addresses this gap by systematically examining the reflection and absorption properties of cement-based EMI shielding composites incorporating CNTs, CFs, and/or GNPs.

## 2. Experimental Procedure

In this study, ordinary Portland cement (OPC) served as the binder material, while sand with particle sizes ranging from 0.17 to 0.7 mm was employed as the aggregate. Silica fume (Elkem Inc., EMS-970, Cliffwood, NJ, USA) was incorporated to enhance the dispersion of nanoparticles within the cement matrix. A water-to-cement ratio of 0.45 was maintained, and a polycarboxylate-based superplasticizer (Dongnam Co., Ltd., FLOWMIX 3000 U, Gyeonggi-do, Republic of Korea) was utilized to achieve the desired flowability for sample fabrication. Three distinct types of conductive fillers, namely, CNTs, CFs, and GNPs, were employed, with detailed specifications available in previous works by the authors. The specific mix proportions employed in this study are summarized in Table 1. As reported in the relevant literature, the incorporation of micron-sized CFs reduce the workability during the fabrication process. Additionally, a large amount of GNPs is necessary to achieve desirable electrical conductivity. Therefore, in this study, the CNT content was fixed at 0.5 wt% for samples containing two types of conductive fillers, while CFs or GNPs were subsequently added to these samples. The sample fabrication procedures followed methodologies outlined in prior investigations.

Prior to determining the levels of incorporated conductive fillers, the electrical conductivity of the composites solely containing CNTs, CFs, or GNPs was evaluated. The selection of each conductive filler content commenced from the percolation threshold region, characterized by a significant decrease in electrical resistivity. The AC conductivity of the produced samples within the X-band frequency range was measured using a PNA network analyzer (N5225B, Keysight, Santa Rosa, CA, USA). Here, the free space EMI shielding measurements were conducted following the standards outlined in the previous study [19].

**Figure 1:**
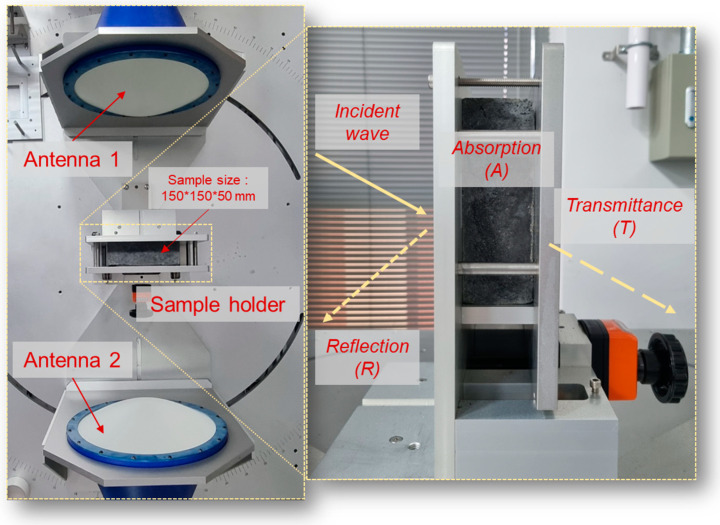
Experimental setup for the EMI shielding measurements.

The EMI shielding performance, represented by four distinct S-parameters (S11, S21, S12, and S22), was assessed according to the configuration depicted in Figure 1. Antenna 1 transmitted electromagnetic waves to antenna 2, enabling the observation of reflection and absorption losses on the sample’s surface and within its interior, respectively. A sample size of 150 × 150 × 50 mm was chosen to optimize the total shielding effectiveness while facilitating focused examination of reflection and absorption losses. The sample was positioned in the center, between the two antennas. Subsequently, the measured S-parameters were converted into reflection, absorption, average power coefficients, and total shielding effectiveness using the following equations:

(1) $$Reflection\left(dB\right)=-10\log\left(1-R\right),\;R={\left|S_{11}\right|}^{2}$$

(2) $$Absorption\left(dB\right)=-10\log\left[T/\left(1-R\right)\right],\;T={\left|S_{12}\right|}^{2}$$

(3) $$Average\;power\;coefficients\left(A\right)=1-R-T$$

(4) $$Total\;EMI\;shielding\;effectiveness\left(dB\right)=Reflection+Absorption$$

## 3. Results and Discussion

**Figure 2:**
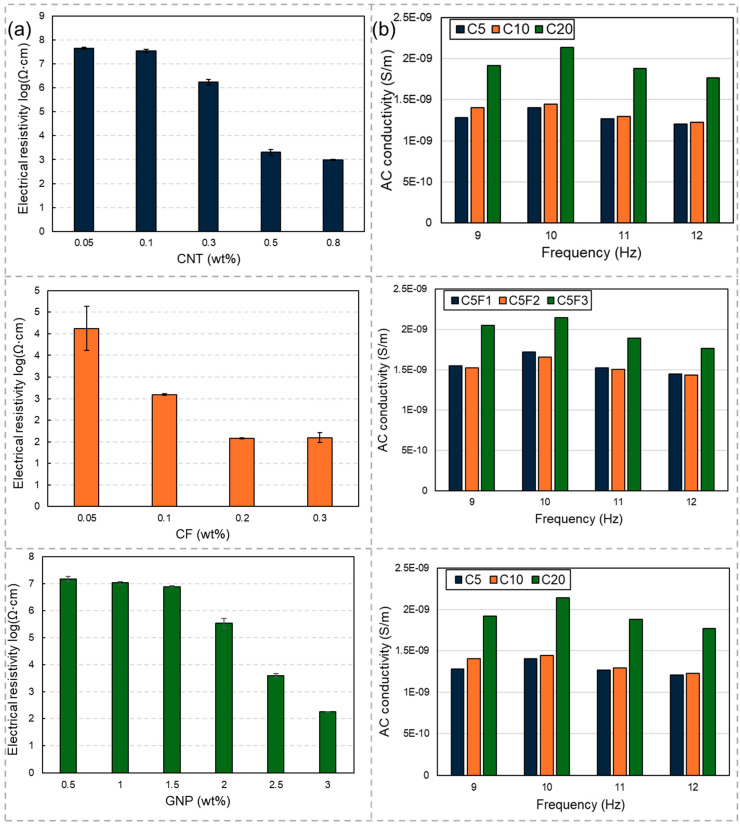
Electrical characteristics: (**a**) electrical resistivities of the samples incorporating solely CNTs, CFs, or GNPs; (**b**) AC conductivity of the samples in the X-band of the frequency range.

Determining the appropriate content of each conductive filler for the EMI shielding composites necessitated an investigation into their percolation thresholds. As noted in previous studies, the percolation threshold represents the point at which the electrical resistivity significantly decreases with increasing amounts of conductive fillers. This method was applied in the current study to assess the percolation thresholds of the composites. Figure 2a illustrates the electrical resistivities of the samples containing solely CNTs, CFs, or GNPs. From the results in Figure 2a, it is evident that the percolation thresholds for CNTs, CFs, and GNPs occur at 0.5 wt%, 0.1 wt%, and 2.5 wt%, respectively. Additionally, previous studies have highlighted the synergistic effects of combining conductive fillers of different sizes, leading to enhanced electrical conductivity and improved functional properties. Consequently, in samples incorporating two different conductive fillers, the content of CNTs was fixed at 0.5 wt%. Figure 2b depicts the AC conductivities of the samples within the X-band frequency range. The permittivity of each sample at different frequencies was measured using a PNA network analyzer and subsequently converted to AC conductivity using Equation (5), as follows:

(5) $$\sigma_{AC}=2\cdot \pi \cdot f\cdot \varepsilon_{0}\cdot \varepsilon^{\prime \prime }$$

where *f*, *ε*_0_, and *ε*″ represent frequency, permittivity of free space, and the imaginary part of permittivity, respectively. In Figure 2b, the synergistic effects resulting from the combination of CNTs with CFs or GNPs are evident. Incorporating 0.1 wt% CFs or 2.5 wt% GNPs alongside 0.5 wt% CNTs yielded a higher AC conductivity than that achieved with 1 wt% CNTs alone. The high level of CNT incorporation in the cement matrix necessitates a high water-to-cement ratio, leading to poor flowability. Thus, incorporating two different fillers can mitigate this issue while simultaneously enhancing the electrical conductivity.

**Figure 3:**
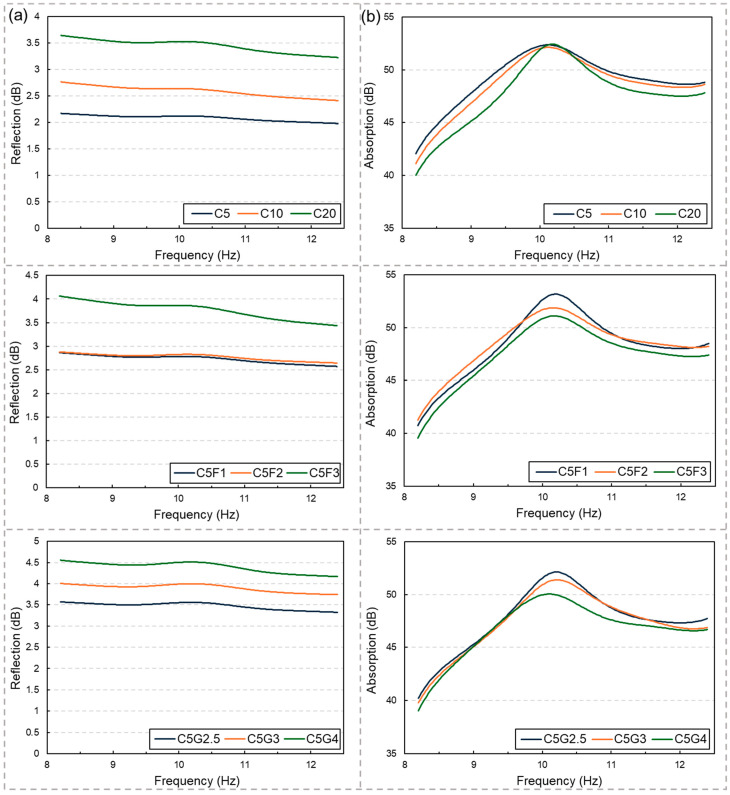
EMI shielding performance of the samples, expressed as (**a**) reflection and (**b**) absorption losses in the X-band of the frequency range.

Figure 3 illustrates the EMI shielding performance, encompassing the reflection and absorption losses. Notably, the sample thickness used in this study (i.e., 50 mm) was sufficient to block approximately 99.999% of the EM wave, emphasizing the investigation’s focus on the ratio of reflection and absorption losses when the majority of the EM wave is intercepted by the samples. In Figure 3a, it can be observed that the reflection loss increases with the incorporation of conductive fillers, irrespective of their types. This trend suggests a proportional relationship between reflection loss and electrical conductivity, consistent with the results in Figure 2. Conversely, the absorption losses, as shown in Figure 3b, did not exhibit significant variations with changes in the electrical conductivity.

**Figure 4:**
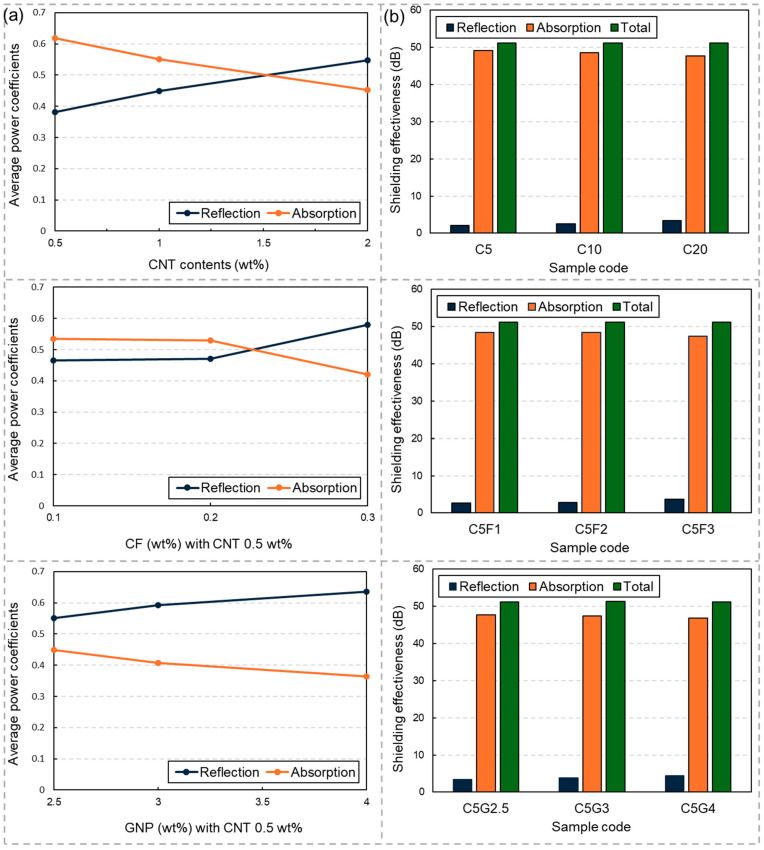
(**a**) Average power coefficients and (**b**) total shielding effectiveness obtained from the EMI shielding experiments.

In addition, the average power coefficients and shielding effectiveness are shown in Figure 4. Notably, the average power coefficients demonstrated distinct trends depending on the conductive filler types. For fiber-type fillers like CNTs and CFs, the average power coefficients of reflection increased while those of absorption decreased with increasing filler content. Moreover, beyond a certain point, the average power coefficient of absorption surpassed that of reflection. Conversely, regardless of the incorporated GNP contents, the average power coefficient of reflection exceeded that of absorption. This phenomenon can be attributed to the plate-type morphology of the GNPs, which effectively intercepted EM waves at the surface of the samples, thereby increasing the reflection loss. Similar observations were reported in the previous studies, indicating that plate-type carbon nanomaterials such as GNPs and graphene enhance EM reflection properties on the surface of EMI shielding composites [20,21]. Thus, it is evident that the type of conductive filler significantly influences EM reflection and absorption properties, even when the total shielding effectiveness remains consistent. Consequently, it is advisable to select the optimal filler contents considering the importance of reflection or absorption losses, as well as the shape or type of the conductive fillers.

## 4. Limitations and Outlook

This study focused on experimentally investigating the influence of different types, sizes, and shapes of conductive fillers on the EMI shielding performance of cement-based composites. In particular, the research evaluated the balance between the reflection and absorption properties to optimize the shielding effectiveness. Based on the experimental results, incorporating two different types of conductive fillers (e.g., CNTs, CFs, or GNPs) was shown to enhance the EMI shielding performance using smaller amounts of the fillers. However, several limitations remain to be addressed in order to increase the applicability of these composites in industrial settings. First, a more comprehensive study, including both the mechanical properties and EMI shielding performance, should be conducted. Many existing studies, including the present study, have focused solely on functional properties without taking into account the mechanical integrity of the composites [7,22,23]. In practical applications, particularly in civil engineering, it is essential to define specific performance targets, ensuring that both the mechanical strength and EMI shielding properties align with the intended use. For instance, while a high level of shielding may be achieved, the mechanical strength must be sufficient to withstand structural loads, especially for applications in infrastructure. Second, the durability and functional stability of the proposed composites under real-world conditions remain unexamined. In many practical applications, the composites may be exposed to harsh environmental conditions such as water ingress, freeze–thaw cycles, or elevated temperatures. These conditions could significantly affect both the mechanical properties and the EMI shielding performance over time [5]. For example, water exposure may affect electrical conductivity by disturbing the conductive network within the cement matrix, while freeze–thaw cycles might lead to crack formation, further compromising both the mechanical and shielding performances. Therefore, long-term studies assessing the stability of these composites under such weathering conditions are crucial. Third, given that these composites are envisioned for use in large-scale civil structures, they should be designed for precast systems. This requires developing methods to achieve high early strengths, ensuring that the composites can be rapidly installed and used prior to completing the standard 28 days of curing typically required for normal concrete. Innovations in fabrication methods tailored to precast systems would also facilitate the use of these composites in industrial settings. Lastly, the high cost of conductive fillers and specialty binders used to fabricate EMI shielding composites presents a significant barrier to their widespread adoption. Current materials, like CNTs, CFs, and GNPs, are expensive, limiting the feasibility of their use in large-scale civil infrastructure. Therefore, research should explore the incorporation of recycled conductive fillers and supplementary cementitious materials (SCMs) as lower-cost alternatives [24,25,26]. Utilizing recycled materials would not only reduce costs but also contribute to sustainability efforts in the construction industry. In summary, while this study demonstrates that combining different types of conductive fillers can enhance the EMI shielding performance of cement-based composites, there is still a need for further research. Investigations into the mechanical properties, durability under environmental stressors, early-strength development for precast applications, and cost-effective alternatives to traditional fillers are necessary to fully unlock the potential of these composites for industrial use. By addressing these challenges, the next generation of cement-based EMI shielding composites could play a vital role in the development of smart and resilient infrastructure.

## 5. Concluding Remarks

This study investigates the impact of different conductive filler types on the ratio of the reflection and absorption properties in cement-based EMI shielding composites. Three distinct fillers, namely, CNTs, CFs, and GNPs, were utilized in the fabrication of these composites. Experimental findings reveal that regardless of the filler type and content, the fabricated samples with a thickness of 50 mm achieved a shielding effectiveness exceeding 50 dB (i.e., 99.999%). However, the type of filler significantly influences the balance between the reflection and absorption losses. Consequently, it is crucial to carefully consider factors such as the significance of reflection or absorption losses, as well as the shapes or types of conductive fillers when selecting the optimal filler contents to fabricate effective cement-based EMI shielding composites.

## Figures and Tables

**Table 1:** Mix proportions of the samples (wt%).

Sample Code	OPC *	Sand	SF	CNT	CF	GNP	Water	SP
C5	100	150	10	0.5	0	0	45	1
C10				1.0				2
C20				2.0				3
C5F1				0.5	0.1	0		1
C5F2					0.2			1.5
C5F3					0.3			2
C5G2.5					0	2.5		1
C5G3						3.0		1
C5F4						4.0		1

* OPC: ordinary Portland cement; SF: silica fume; CNTs: carbon nanotubes; CFs: carbon fibers; GNPs: graphene nanoplatelets; SP: superplasticizer.

## Data Availability

The original contributions presented in the study are included in the article, further inquiries can be directed to the corresponding authors.
